# Supplementary material for: Insights into the Interaction Mechanisms of the Proviral Integration Site of Moloney Murine Leukemia Virus (Pim) Kinases with Pan-Pim Inhibitors PIM447 and AZD1208: A Molecular Dynamics Simulation and MM/GBSA Calculation Study
Source: Int J Mol Sci. 2019 Oct 30;20(21):5410. doi: 10.3390/ijms20215410 (PMC6862308; doi:10.3390/ijms20215410)
Supplement: Supplementary file 1 [file ijms-20-05410-s001.zip › ijms-615467-SI.docx]

Supplementary Material

**Insights into the interaction mechanisms of the proviral integration site of Moloney murine leukemia virus (Pim) kinases with pan-Pim inhibitors PIM447 and AZD1208: A molecular dynamics simulation and MM/GBSA calculation study**

Qingqing Chen ^1,#^, Yan Wang^1,#^, Shanshan Shi ^1^, Kaihang Li ^1^, Ling Zhang ^1^ and Jian Gao ^1,*^

^1^Jiangsu Key Laboratory of New Drug Research and Clinical Pharmacy, Xuzhou Medical University, Xuzhou, Jiangsu 221004, China;

*****Correspondence: gaojian@xzhmu.edu.cn; Tel.: +86-0516-8326-2137

^#^ Qingqing Chen and Yan Wang are contributed equally to this work.

**

**

**Figure S1** RMSF of the backbone atoms of protein versus the residue numbers of Pims in six systems. The purple dotted lines indicate the key residues for inhibitor binding.

**Table S1.** Binding free energies of six Pim/ligand systems calculated in MM/GBSA (kcal/mol).

| System | Δ*E*_vdw_ | Δ*E*_ele_ | ΔG_GB_ | ΔG_SA_ | TΔS | ΔG_bind_ | K_i_ (nM) |
| --- | --- | --- | --- | --- | --- | --- | --- |
| Pim1/GDC-0339 | -42.2±2.71 | -15.33±3.78 | 19.85±3.45 | -6.55±0.22 | -12.56±2.28 | -31.51±2.86 | 0.03 |
| Pim1/Hispidulin | -34.45±2.91 | -12.38±5.31 | 18.68±4.50 | -4.87±0.19 | -12.24± 2.71 | -20.77±2.73 | 2710 |
| Pim2/GDC-0339 | -35.76±2.91 | -18.66±3.19 | 22.65±2.73 | -6.84±0.17 | -10.14±2.28 | -28.47±2.80 | 0.1 |
| Pim2/Hispidulin | -41.62±2.49 | -15.12±3.43 | 23.37±3.25 | -5.17±0.15 | -13.35± 2.45 | -25.19±2.54 | - |
| Pim3/ GDC-0339 | -42.2±2.71 | -17.8±3.22 | 22.3±2.68 | -6.71±0.16 | -11.27± 2.63 | -33.13±2.42 | 0.02 |
| Pim3/Hispidulin | -35.15±3.28 | -24.80±4.48 | 29.05±3.39 | -5.18±0.10 | -12.36± 2.36 | -23.71±2.65 | - |
